# Rapid high throughput SYBR green assay for identifying the malaria vectors *Anopheles arabiensis*, *Anopheles coluzzii* and *Anopheles gambiae s*.*s*. *Giles*

**DOI:** 10.1371/journal.pone.0215669

**Published:** 2019-04-19

**Authors:** Joseph Chabi, Arjen Van’t Hof, Louis K. N’dri, Alex Datsomor, Dora Okyere, Harun Njoroge, Dimitra Pipini, Melinda P. Hadi, Dziedzom K. de Souza, Takashi Suzuki, Samuel K. Dadzie, Helen P. Jamet

**Affiliations:** 1 Parasitology Department, Noguchi Memorial Institute for Medical Research, University of Ghana, Legon, Accra, Ghana; 2 Liverpool School of Tropical Medicine, Liverpool, United Kingdom; 3 Kemri-Wellcome Trust Research Programme, Kilifi, Kenya; 4 Vestergaard Frandsen East Africa, Nairobi, Kenya; 5 Section Environmental Parasitology, Kobe-Tokiwa University, Nagata-Ku, Japan; 6 Vestergaard regional office, Washington DC, United States of America; University of Helsinki, FINLAND

## Abstract

The *Anopheles gambiae sensu lato* species complex consists of a number of cryptic species with different habitats and behaviours. These morphologically indistinct species are identified by chromosome banding. Several molecular diagnostic techniques for distinguishing between *An*. *coluzzii* and *An*. *gambiae* are still under improvement. Although, the current SINE method for identification between *An*. *coluzzii* and *An*. *gambiae* works reliably, this study describes a refinement of the SINE method to increase sensitivity for identification of *An*. *coluzzii*, *An*. *gambiae* and *An*. *arabiensis* based on amplicon dissociation curve characteristics. Field-collected samples, laboratory-reared colonies and crossed specimens of the two species were used for the design of the protocol. *An*. *gambiae*, *An*. *coluzzii*, and hybrids of the two species were sampled from Ghana and *An*. *arabiensis* from Kenya. Samples were first characterised using conventional SINE PCR method, and further assayed using SYBR green, an intercalating fluorescent dye. The three species and hybrids were clearly differentiated using the melting temperature of the dissociation curves, with derivative peaks at 72°C for *An*. *arabiensis*, 75°C for *An*. *gambiae* and 86°C for *An*. *coluzzii*. The hybrids (*An*. *gambiae* / *An*. *coluzzii*) showed both peaks. This work is the first to describe a SYBR green real time PCR method for the characterization of *An*. *arabiensis*, *An*. *gambiae* and *An*. *coluzzii* and was purposely designed for basic melt-curve analysis (rather than high-resolution melt-curve) to allow it to be used on a wide range of real-time PCR machines.

## Introduction

The *Anopheles gambiae* sensu lato (*An*. *gambiae* s.l.) complex comprises at least seven mosquito species originally defined by polytene chromosome analysis [1, 2]. All the *An*. *gambiae* s.l. species are currently identified by PCR-diagnostic assays based on specific DNA nucleotide differences in the intergenic spacer (IGS) of the ribosomal DNA (rDNA) [3–6]. Detailed analyses of the IGS region of rDNA further revealed nucleotide substitutions that differentiated between the two forms within the *Anopheles gambiae s*.*s* previously designated as S and M molecular forms [7], and were recently named *An*. *gambiae* and *An*. *coluzzii* [8]. These two species can be identified by PCR and gel electrophoresis showing the presence or absence of a diagnostic Short Interspersed Nuclear Element (SINE) on the X-chromosome. [9].

Hybrid forms of *An*. *gambiae* and *An*. *coluzzii* have been identified [10, 11]. Several countries have reported high proportion of hybrid population within wild adult mosquito collections. In Gambia, *An*. *gambiae* / *An*. *coluzzii* hybrids were identified from a number of sites at frequencies as high as 16.7% [12] while in Guinea-Bissau, over 20% of the individuals assayed were hybrids and later, more than 40% were observed in the same country and in Senegal [13–15]. However, correct hybrid species identification [16] and additional data on hybrid species distribution and potential survival rates of wild populations is still needed. It has been described that the first progeny (F1) of hybrids were fully fertile [17], and recent laboratory-based crossing experiments have shown that the hybrids of *An*. *gambiae* / *An*. *coluzzii* can be maintained over several generations [18] instigating the need to clearly differentiate hybrid *An*. *gambiae* / *An*. *coluzzii* within wild population of *An*. *gambiae* s.l.

*Anopheles gambiae* and *An*. *coluzzii* are characterised by a high degree of gene flow restriction, low level of genetic differentiation, and a largely overlapping geographical and temporal distribution [19]. Furthermore, the two species, together with *An*. *arabiensis*, can live in sympatry but show different living characteristics such as insecticide resistance profile with varying resistance allele distributions [20]. Therefore, the correct identification of *An*. *arabiensis*, *An*. *gambiae* and *An*. *coluzzii* mosquitoes constitutes an integral part of malaria vector control programmes, and insecticide resistance management.

A commonly used method for differential identification of *An*. *arabiensis*, *An*. *gambiae* and *An*. *coluzzii* involves a combination of protocols established by Scott *et al*. and Fanello *et al*.[4, 11, 21]. These methods are based on PCR-Restriction Fragment Length Polymorphism (PCR-RFLP) and make use of the presence of nucleotide substitutions within the 28S coding region, and part of the IGS region of rDNA [11]. More recently a Short Interspersed Nuclear Element (SINE) insertion (S200 X6.1) on the X chromosome of *An*. *gambiae* has been found to be fixed in all *An*. *coluzzii* and absent in *An*. *gambiae* and *An*. *arabiensis* [10]. Additionally, a 26 bp deletion in the same region defines *An*. *arabiensis*, allowing for the development of a novel PCR diagnostic assay that differentiates the three species [10]. Briefly, primers that flank the S200 X6.1 insertion were designed to amplify the genomic DNA isolated from *Anopheles gambiae s*.*s*. specimen. The PCR products are run on an agarose gel, and individuals (*An*. *coluzzii*) with the S200 X6.1 insertion show a single band at 479 bp while individuals (*An*. *gambiae*) with no S200 X6.1 insertion give a 249 bp band, and *An*. *arabiensis* 223 bp as a result of the deletion [10]. Either method for the identification of *An*. *arabiensis*, *An*. *gambiae* and *An*. *coluzzii* requires post-PCR analysis by gel electrophoresis, which is less sensitive and laborious than melt-curve analysis.

SYBR green DNA-based Real-Time PCR systems provide a good alternative to fluorescent probe-based Real-Time PCR techniques and are based on ability of SYBR green to produce a 100-fold increase in fluorescence when bound to double-stranded DNA. Even though SYBR green binds non-specifically to nucleic acids, the fluorescent signal produced when in complex with DNA is directly proportional to the length and amount of DNA copies synthesized during the reaction, making this technique very sensitive [22] and very precise when diagnostic primer sets are used.

The aim of this study is to demonstrate a time-efficient, highly sensitive and specific SYBR green-based real-time PCR diagnostic assay that differentiates between *An*. *arabiensis*, *An*. *coluzzii* and *An*. *gambiae*.

## Materials and methods

### Mosquito samples and DNA extraction

*Anopheles gambiae* mosquito samples were obtained from Vestergaard-NMIMR Vector Labs (VNVL). These consisted of more than 200 samples selected from the standard susceptible Kisumu strain originally from Kenya, and *An*. *gambiae* Tiassalé, a resistant strain from the village of Tiassalé in Côte d’Ivoire maintained in the VNVL insectary since 2010. Hundred and ninety *An*. *coluzzii* were collected from Okyereko, a rice irrigation field in the Central region of Ghana [23–25]. Hybrid *An*. *gambiae* / *An*. *coluzzii* (75 samples) were obtained from laboratory crossing of either *An*. *gambiae* Kisumu and *An*. *coluzzii* or *An*. *gambiae* Tiassalé and *An*. *coluzzii*. Fifty *An*. *arabiensis* samples were obtained from field collection in Kenya. The study was initiated at Noguchi Memorial Institute for Medical Research (NMIMR) in Ghana and the primer design, optimization, validation and high-throughput species identification were performed at Liverpool School of Tropical Medicine (LSTM), UK.

Whole mosquito DNA extraction was performed using a simplified version of the protocol designed by Collins and colleagues [26]. A single mosquito was homogenized in a 1.5 ml Eppendorf tube containing 200 μl of CTAB buffer and incubated at 65°C in a water bath for 5 minutes. Two hundred microliters (200 μl) of chloroform were added to the homogenate, mixed by inversion and centrifuged for 5 minutes at 12000 rpm 25°C. The supernatant was pipetted into new 1.5 ml Eppendorf tubes. 200 μl of isopropyl alcohol was added, mixed by inversion and then centrifuged at 12000 rpm for 15 minutes. The supernatant was then discarded gently and the DNA pellet was thereafter purified with 70% ethanol, dried overnight, and reconstituted in 20 μl of DNAse free water.

### Mosquito species identification

*Anopheles arabiensis and An*. *gambiae* s.s. species were first determined using established protocols [4, 11]. Conventional PCR assays were performed with a 1 in40 dilution of the reconstituted DNA solution obtained from a single mosquito. PCR products were run on 2% agarose gels, stained with ethidium bromide and then visualized using UV Trans-illuminator (BioDoc-It Imaging System, Upland, USA).

SINE PCR was performed using primers designed by Santolamazza *et al*. [10] for the identification of *An*. *arabiensis*, *An*. *coluzzii* and *An*. *gambiae* using the primers; F6.1a (TCGCCTTAGACCTTGCGTTA) and R6.1b (CGCTTCAAGAATTCGAGATAC). Total PCR reaction volume of 25 μl containing 4 μl of 1 in 40 dilution of genomic DNA (used as template), 1 μl each of 10 μM of both forward and reverse primers, 6.5 μl of nuclease-free water and 12.5 μl of GoTaq master mix (Promega, Madison, WI, USA). Reaction conditions used were 94°C for 10 minutes; followed by 35 cycles of 94°C for 30 seconds, 59°C for 30 seconds72°C for 1 minute; and a final extension at 72°C for 10 minutes. PCR products were run on 2% agarose gels, stained with ethidium bromide and thereafter visualized using UV Trans-illuminator.

### SYBR green-based Real-Time PCR for species identification

This designed melt curve method to distinguish between *An*. *arabiensis*, *An*. *gambiae* and *An*. *coluzzii* uses the diagnostic Short Interspersed Nuclear Element (SINE200). The original S200 X6.1 primers designed by [10] were unsuitable for melt curve analysis, firstly because they produce overlapping melt-peaks for *An*. *coluzzii* and *An*. *gambiae* /*An*. *coluzzii* hybrids and secondly they give indistinct melt peaks for *An*. *gambiae* and *An*. *arabiensis*. New primers were designed based on amplicon length and G/C content to produce distinct melt-curves for the three sibling species and the hybrids. A universal forward primer (SINE200Fa 5’-ATTGCTACCACCAAAATACATGAAA-3’) matching all three species is combined with *An*. *gambiae* / *An*. *coluzzii* specific reverse (SINE200Rd 5’-GGGGGGGGGAATAATAAGGAACTGCATTTAAT-3’) and for *An*. *arabiensis* reverse (SINE200Re 5’- GGATGTCTAATAGTCTCAATAGATG -3’).

SINE200Rd, the reverse primer for *An*. *gambiae* and *An*. *coluzzii* has a G8 stretch (GGGGGGGG) added to the 5’ end of the primer (as underlined) to increase the amplicon melting temperature (Tm), preventing the melt profiles of *An*. *gambiae* and *An*. *arabiensis* to overlap. SINE200Rd incorporates the SINE200 transposon in *An*. *coluzzii* into the amplicon and gives a distinct high Tm. The specificity of the *An*. *arabiensis* primer SINE200Re is based on only two mismatches with *An*. *gambiae*. Both mismatches are conveniently on the 3' end of the primer though a sufficiently high annealing temperature effectively prevents amplification of *An*. *gambiae*. The amplicon sizes are 60 bp, 103 bp and 333 bp for *An*. *arabiensis*, *An*. *gambiae* and *An*. *coluzzii* respectively.

A total of sixty (60) samples including 10 *An*. *arabiensis*, 20 of *An*. *gambiae*, 14 *An*. *coluzzii*, and 15 hybrids randomly selected among the individuals characterized by SINE PCR, and one artificial hybrid (pooled DNA of *An*. *gambiae-An*. *coluzzii*) were analysed using the designed melt-curve protocol.

A total reaction volume of 10 μl mixture was prepared by combining 3.75 μl of Nuclease-free PCR grade water, 5 μl of Luna Universal qPCR master mix (New England Biolabs, Ipswich, Ma, USA), 0.25 μl of each (10 μM) SINE200Fa, SINE200Rd and SINE200Re primers, and 0.5 μl mosquito DNA template (Table 1).

**Table 1:** SYBR green master mix.

	1X (μl)
**Nuclease free water**	3.75
**Luna Universal qPCR Master Mix**	5
**SINE200Fa (10 μM)**	0.25
**SINE200Rd (10 μM)**	0.25
**SINE200Re (10 μM)**	0.25
**Genomic DNA template**	0.5
**Total**	10

The samples were run on AriaMx Real-time PCR system (Agilent, Santa Clara, Ca, USA) using the quantitative PCR DNA binding dye including standard melt curve program and 520 nm wavelength filter (FAM). Annealing temperature (Ta) and cycle number were optimised to eliminate non-target background melt-peaks. The optimized cycling conditions involved a denaturation of 95°C for 60 seconds followed by 33 cycles of 95°C for 15 seconds, 60°C for 20 seconds, and 72°C for 10 seconds with a final dissociation step of 95°C for 60 seconds, 55°C for 30 seconds and a melt ramp up to 95°C with 0.5°C increments.

The robustness of the assay was tested using SYBR green-based alternative reagents and an alternative real-time PCR machine. Eight samples of each group (*An*. *arabiensis*, *An*. *gambiae*, *An*. *coluzzii*, and hybrid) were repeated with Luna Universal qPCR Master Mix and with Brilliant III Ultra-Fast SYBR Green Low ROX QPCR Master Mix (Agilent) on a Stratagene Mx3005P real-time PCR machine (Agilent). Thermal cycling conditions and PCR mix were identical to those used for AriaMX. Furthermore, 88 samples from Sudan and 87 from Tanzania were run in both machines to check for consistency and degree of shift between machines.

The species identification was automated using a “nested if” statement in Microsoft Excel which assigns the melt peak temperatures to pre-defined temperature windows corresponding with the different species (S1 Text).

The peak temperature criteria are: >85°C = AC = *An*. *coluzzii*, between 85°C and 74°C = AG = *An*. *gambiae*, <74°C = AA = *An*. *arabiensis*, both >85°C and between 85°C and 74°C = HY = *An*. *gambiae /An*. *coluzzii* hybrid. This automation prevents the need for manually scoring species as is the case for gel-based species ID, thereby reducing time and scoring errors. A large number of samples (1075 mosquitoes) collected in western Kenya between 2011 and 2015 whose species ID was previously assigned using the 28S IGS gel-based method [11] were re-examined using the melt-curve technique. A subset of 24 individuals identified as *An*. *gambiae* with the 28S method and *An*. *arabiensis* according to the melt-curves plus all 10 *An*. *arabiensis* re-scored as *An*. *gambiae* were repeated with both the gel-based SINE200 method [10] and the 28S method [11]

The SINE200 region was later investigated in-silico to predict the performance of the assay for mosquitoes collected throughout Sub-Saharan Africa. For *An*. *gambiae* and *An*. *coluzzii*, the primer binding sites were examined in the phase 1 data of the *Anopheles gambiae* 1000 genome project (https://www.malariagen.net/apps/ag1000g/), covering West and East African populations. To predict whether the method can also be used for West-African *An*. *arabiensis*, sequence runs for 9 *An*. *arabiensis* individuals from Cameroon, and 10 from Burkina Faso were examined (S1 Table). Raw sequence reads were mapped against the SINE200 region of the AaraD1 assembly using Geneious 10 (Biomatters Ltd.) and examined for polymorphisms.

## Results

### Species identification

#### SINE PCR

**Fig 1:**
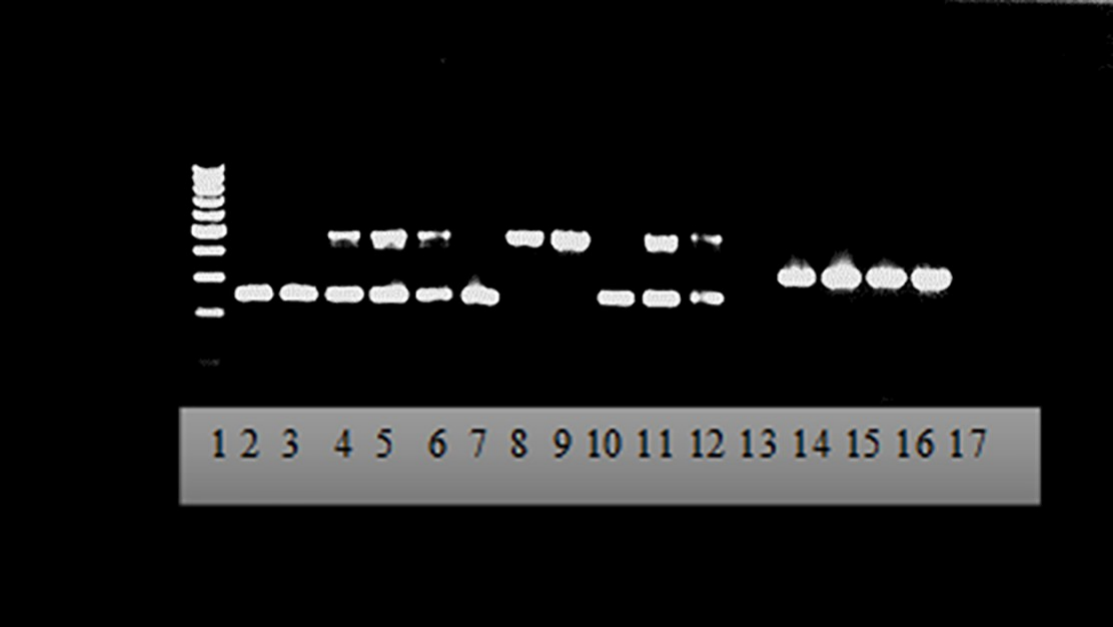
2.0% agarose gel for SINE PCR showing *An*. *arabiensis*, *An*. *gambiae* and *An*. *coluzzii*. Lane 1: molecular weight ladder (100 bp); Lane 2; 3 & 10: An. gambiae, Lane 4–6 & 11–12: hybrids An. coluzzii / An. gambiae; Lane 8 & 9: An. coluzzii, Line 14–17: An. arabiensis. DNA fragments of 315 bp for An. arabiensis, 249 bp (An. gambiae) and 479 bp (An. coluzzii). Hybrid showed both band sizes at 249 bp and 479 bp.

The sixty samples selected, including 10 *An*. *arabiensis*, 20 *An*. *gambiae*, 14 *An*. *coluzzii* and 15 hybrids of both species and one pooled artificial hybrid were identified using SINE PCR. Gel electrophoresis analyses of SINE PCR products showed distinct diagnostic bands corresponding to each species. *Anopheles arabiensis*, *An*. *coluzzii* and *An*. *gambiae* showed specific band sizes of 315 bp, 479 bp and 249 bp respectively, while the *An*. *gambiae*/*An*. *coluzzii* hybrids showed both amplicon sizes (Fig 1).

#### SYBR green real-time PCR

**Fig 2:**
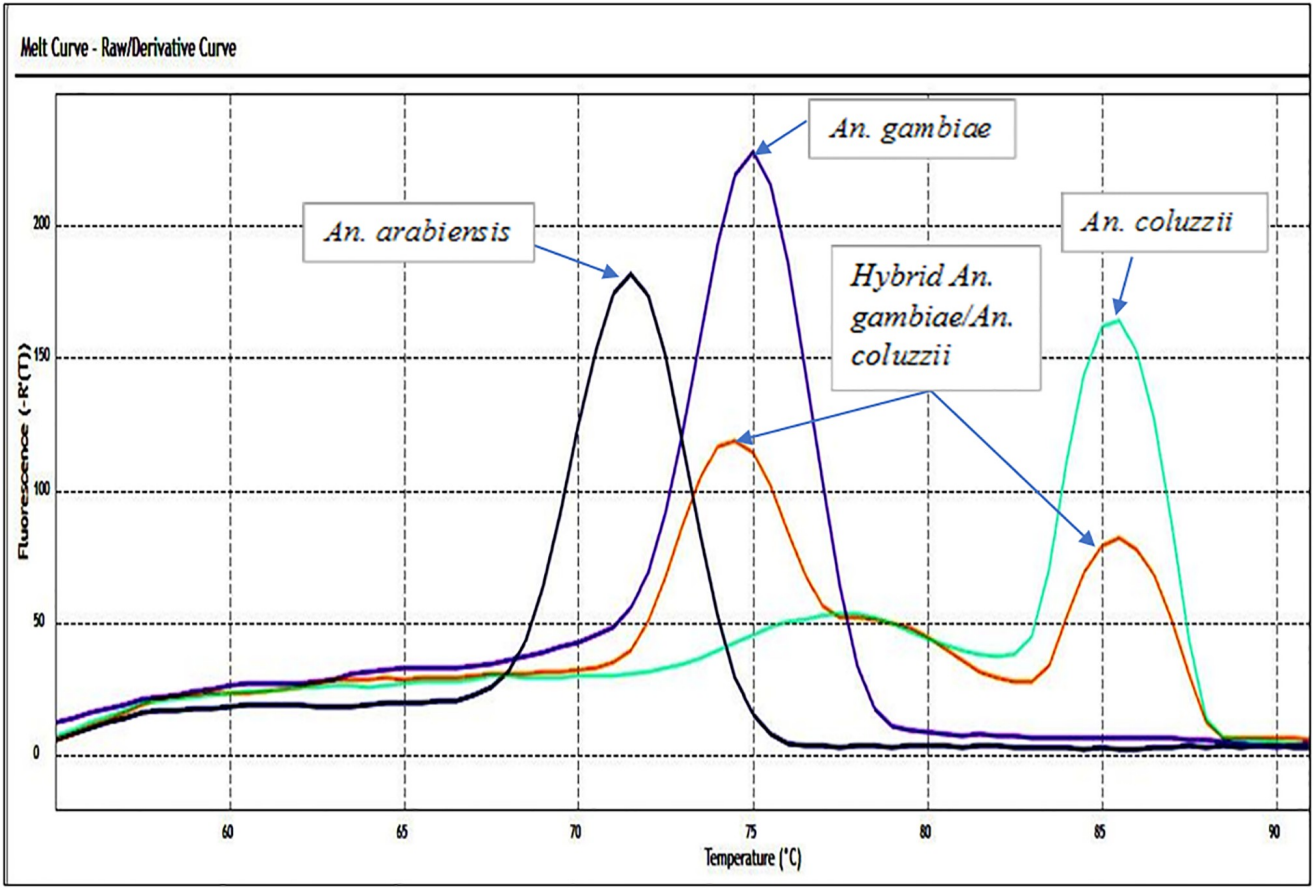
Dissociation curves of *all species* (*An*. *gambiae*, *An*. *coluzzii*, hybrid *An*. *gambiae / An*. *coluzzii* and *An*. *arabiensis*) using SYBR green high throughput methods.

The identification of all the species and hybrid was based on specific melting temperatures (Tm) from the dissociation curves (Fig 2). *Anopheles arabiensis* showed a single peak at an average temperature of 72°C, *An*. *gambiae* at 75°C; whilst *An*. *coluzzii* peaks at 86°C (Fig 2). The hybrid showed the expected 86°C melting peak for *An*. *coluzzii* and shifted slightly for *An*. *gambiae* (74°C) instead of the expected 75°C. The pooled *An*. *gambiae—An*. *coluzzii* DNA produced a melt-curve identical to hybrids. Results were consistent between the two Real-time PCR machines and between alternative SYBR green qPCR master mixes, except for a slight shift (±1°C) in species-specific melt peaks between the machines. This “machine effect” demonstrates the importance of using positive controls for the three species to calibrate the species-specific peak temperature intervals when using different real-time machines (S1–S3 Figs).

Re-evaluation of 1075 mosquitoes from western Kenya previously screened with 28S IGS revealed an inconsistency rate of 36% between the gel-based method and dissociation curve method. Out of 354 mosquitoes initially scored as *An*. *arabiensis*, 10 were identified as *An*. *gambiae* using the melt-curve technique, and 373 out of 721 assigned *An*. *gambiae* produced an *An*. *arabiensis*-specific melt peak. To investigate whether the inconsistently was caused by wrongly scored 28S IGS gels or by unreliable melt curves, a subset of inconsistent individuals was re-examined using both SINE200 and the 28S IGS gel methods allowing the gels to run sufficiently long to reliably distinguish the diagnostic bands (described in the Method section). The re-examined gel-based scores were now fully consistent with the melt-curve species identifications. Only long gel electrophoresis times showed distinct differences in band sizes between *An*. *gambiae* and *An*. *arabiensis* for both methods (S4 Fig).

In-silico validation of the assay showed two SNPs coinciding with SINE200Fa primer binding site and one in the SINE200Rd binding site based on the *Anopheles gambiae* 1000 genome data. The allele frequencies of these SNPs are 0.07%, 0.07%, and 0.20% and are therefore expected to have a negligible effect on assay performance. The primer sites as well as the sequence between them (i.e. the whole amplicon region) was completely conserved and identical to the AaraD1 assembly in the West-African *An*. *arabiensis* sequence runs. The in-silico validation therefore shows that the assay is expected to work across a wide range of populations and for all three species.

## Discussion

Rapid and reliable identification of species and sub-species of malaria vector populations is an important part of malaria vector control programmes. PCR-RFLP and SINE PCR methods have been developed for this purpose and have both shown to successfully identify *An*. *arabiensis*, *An*. *gambiae* and *An*. *coluzzii* out of the complex of eight species [4, 10, 11]. However, both methods involve at least two PCR steps with gel staining that require precision and time to identify the species. In addition, inconsistent identification of *An*. *gambiae*, *An*. *coluzzii* and their hybrids has been reported by either PCR using form-specific primers or PCR-RFLP genotyping carried out in different laboratories [16]. Mis-identification of *An*. *gambiae* vs *An*. *arabiensis* based on gel-bands is even more likely since both the SINE200 and the 28S IGS techniques produce similar-sized amplicon sizes (223 vs 249 bp and 315 bp vs 390 bp respectively) which only separate when gels are run sufficiently long (the *An*. *gambiae* /*An*. *coluzzii* distinctive HhaI digest [11] is usually not performed in Eastern Africa). The 36% error rate in western Kenyan samples clearly demonstrates the risk of mis-interpreting bands on gels, and consequently the value of the melt-curve approach described here. From Scott *et al*. 1993 to date, SINE PCR has shown greater reliability among all the protocols allowing the differentiation of *An*. *gambiae* and *An*. *coluzzii*. Moreover, each species identification protocol allows partial identification of the whole *An*. *gambiae* complex (Table 2). In addition to the benefits outweighed given the fastest and more reliable results, the current SYBR green melt-curve technique allows the full identification of the three main malaria vectors, *An*. *gambiae*, *An*. *coluzzii* and *An*. *arabiensis* species simultaneously.

**Table 2:** Summary of the different protocols for the identification of *An*. *gambiae* s.l. complex.

METHODS	STEPS		DIAGNOSIS	Identification Methods	REFERENCE
	STEP 1	STEP 2			
Sequencing ITS2 region	ITS2 (Internal transcribed spacers) & COI (cytochrome oxidase sub unit 1)		*An*. *gambiae s*.*s*. *An*. *quadriannulatus*, *An*. *arabiensis & An*. *funestus s*.*s*	DNA sequence	Lobo *et*.*al* 2015
MULTIPLEX RT-PCR	Taqman SNP genotyping + LNA(locked nucleic acid)		*An*. *gambiae* s.s., *An*. *melas*, *An*. *merus*, *An*. *quadriannulatus & An*. *arabiensis*	Fluorescent / Melting temperature	Bass *et al*, 2008.
SINE PCR*	Conventional PCR		*An*. *gambiae*, *An*. *coluzzii & An*. *arabiensis*	Gel electrophoresis	Santolamazza *et*.*al* 2008
Taqman RT-PCR	RT-PCR 28S IGS Taqman	Taqman SNP genotyping *using Walker et al*, *2007*	*An*. *gambiae* s.s., *An*. *melas*, *An*. *merus*, *An*. *quadriannulatus* & *An*. *arabiensis*	Fluorescent	Bass *et al*, 2007
RT-PCR	Taqman SNP genotyping		*An*. *gambiae s*.*s*, *An*. *arabiensis An*. *quadriannulatus* as a false positive for *An*. *gambiae*	Fluorescent/Melting temperature	Walker *et*.*al* 2007
Multiplex PCR*	Conventional PCR		*An*. *quadriannulatus*, *An*. *melas/An*. *merus*, *An*. *gambiae*, *An*. *arabiensis & An*. *coluzzii*	Gel electrophoresis	Wilkins et al, 2006
Multiplex PCR	Conventional PCR		*An*. *quadriannulatus*	Gel electrophoresis	Fettene *et*.*al* 2003
Normal PCR	Conventional PCR		*An*. *quadriannulatus B*	Gel electrophoresis	Fettene *et al*, 2002
PCR-RFLP*	Conventional PCR	RFLP	*An*. *gambiae*, *An*. *coluzzii*, *An*. *melas*, *An*. *merus*, *An*. *quadriannulatus* & *An*. *arabiensis*	Gel electrophoresis	Fanello *et al*, 2002
Normal PCR*	Conventional PCR		*An*. *gambiae*, *An*. *coluzzii*, *hybrid & Bamako form*	Gel electrophoresis	Favia *et al*, 2001
PCR-RFLP*	Conventional PCR	RFLP; 28S IGS using Hha1 or Tru91 enzyme	*An*. *gambiae*, *An*. *coluzzii & Bamako form*	Gel electrophoresis	Favia *et al*, 1997
Normal PCR	Conventional PCR		*An*. *bwambae*	Gel electrophoresis	Townson *et al* 1994
Normal PCR	Conventional PCR		*An*. *gambiae s*.*s*, *An*. *melas*, *An*. *merus*, *An*. *arabiensis & An*. *quadriannulatus*	Gel electrophoresis	Scott et.al 1993

*Represents the protocols enabling the identification of An. gambiae and An. coluzzii

The aim of this study was to demonstrate a SYBR green, rapid, high throughput assay capable of identifying *An*. *arabiensis*, *An*. *gambiae* and *An*. *coluzzii* with high specificity and precision. The high throughput SYBR green rapid assay described here shows great specificity. Though the existing protocols have proven to be useful tools for species identification, this new high throughput assay does not require post PCR analyses such as restriction enzyme digestion and gel electrophoresis associated with PCR-RFLP and SINE PCR [10, 11].

Hence, this new assay showed the first use of SYBR green designed set of primer sequences to distinguish *An*. *arabiensis*, *An*. *gambiae* and *An*. *coluzzii* by Real-Time melt-curve analysis. The newly developed method together with Taqman qPCR methods [27] will allow complete characterization of mosquito specimen into species using Real-Time PCR. Compared to the existing method, this assay is faster, and the closed-tube reaction (without gel electrophoresis) reduces the risk of post-PCR contamination. The assay designed for basic melt-curve analysis (rather than high-resolution melt-curve) allows the use on a wide range of real-time PCR machines.

## Conclusion

The SYBR green real time PCR techniques showed an additional option for the characterization of both sibling species and *An*. *arabiensis* while all the other real time PCR protocol of species differentiation could not allow the characterization of the sub-species such as *An*. *gambiae* and *An*. *coluzzii*. The assay designed in this study is a new tool to help researchers and particularly malaria vector control entities to identify clearly the subspecies within the *An*. *gambiae* s.l. complex in the context where each of the species has unique behaviour and impact on malaria.

## Supporting information

S1 Fig Species ID Melt-curve of Tanzania samples using Mx3005P real time PCR machine. (TIF)

S2 Fig Species ID Melt-curve of Sudan samples using AriaMx real time PCR machine. (TIF)

S3 Fig Dissociation curves of *all species* using SYBR green high throughput methods with two different mixes and Mx3005P real time PCR machine. **A**: Dissociation curves using Brilliant III Ultra-Fast SYBR Green Low ROX QPCR Master Mix. **B**: Dissociation curves using Luna Universal qPCR Master Mix “Any difference was observed using the same machine and different SYBR green master mix”.
(TIF)

S4 Fig Gel images of re-examined individuals with inconsistent species assignment. “Samples with inconsistent species ID scores between the 28S gel analysis and the melt-curve technique were re-examined using both the SINE method and the 28S methods. A subset of 24 out of 373 potentially misidentified *An*. *arabiensis*, and all 10 potentially misidentified *An*. *gambiae* were included. The gel pictures presented here show 5 *An*. *gambiae* (Line 1–5) and 16 *An*. *arabiensis* (Line 6–21) according to the melt-curves (but scored different previously). Pictures were taken at two intervals; 33 minutes and 80 minutes for the 28S PCRs and 40 and 120 minutes for the SINE200 PCRs (electrophoresis at 120 volts). Size differences are very similar for *An*. *gambiae* and *An*. *arabiensis* at the shorter run times, making it difficult to reliably assign species ID. The longer gel runs confirm the melt-curve species ID”.
(TIF)

S1 Text “Nested IF” statement to assign species ID from melt-curve data. (DOCX)

S1 Table Details of the West-African *An*. *arabiensis* sequence runs used to validate the assay in-silico. (XLSX)
