# Supplementary figures and images for: Rapid high throughput SYBR green assay for identifying the malaria vectors Anopheles arabiensis, Anopheles coluzzii and Anopheles gambiae s.s. Giles
Source: PLoS One. 2019 Apr 19;14(4):e0215669. doi: 10.1371/journal.pone.0215669 (PMC6474623; doi:10.1371/journal.pone.0215669)

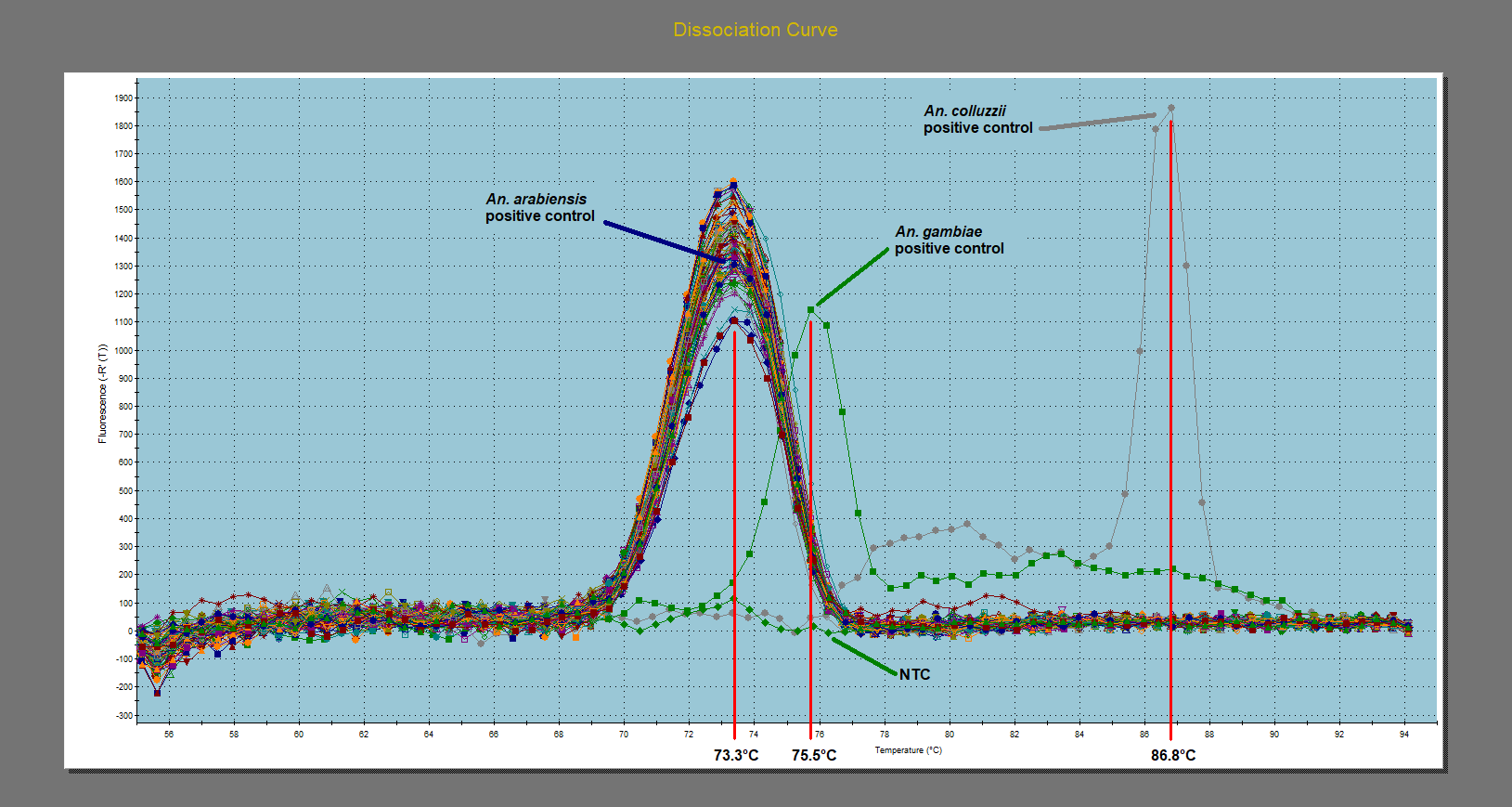

Supplement: S1 Fig — (TIF) [file pone.0215669.s001.TIF]

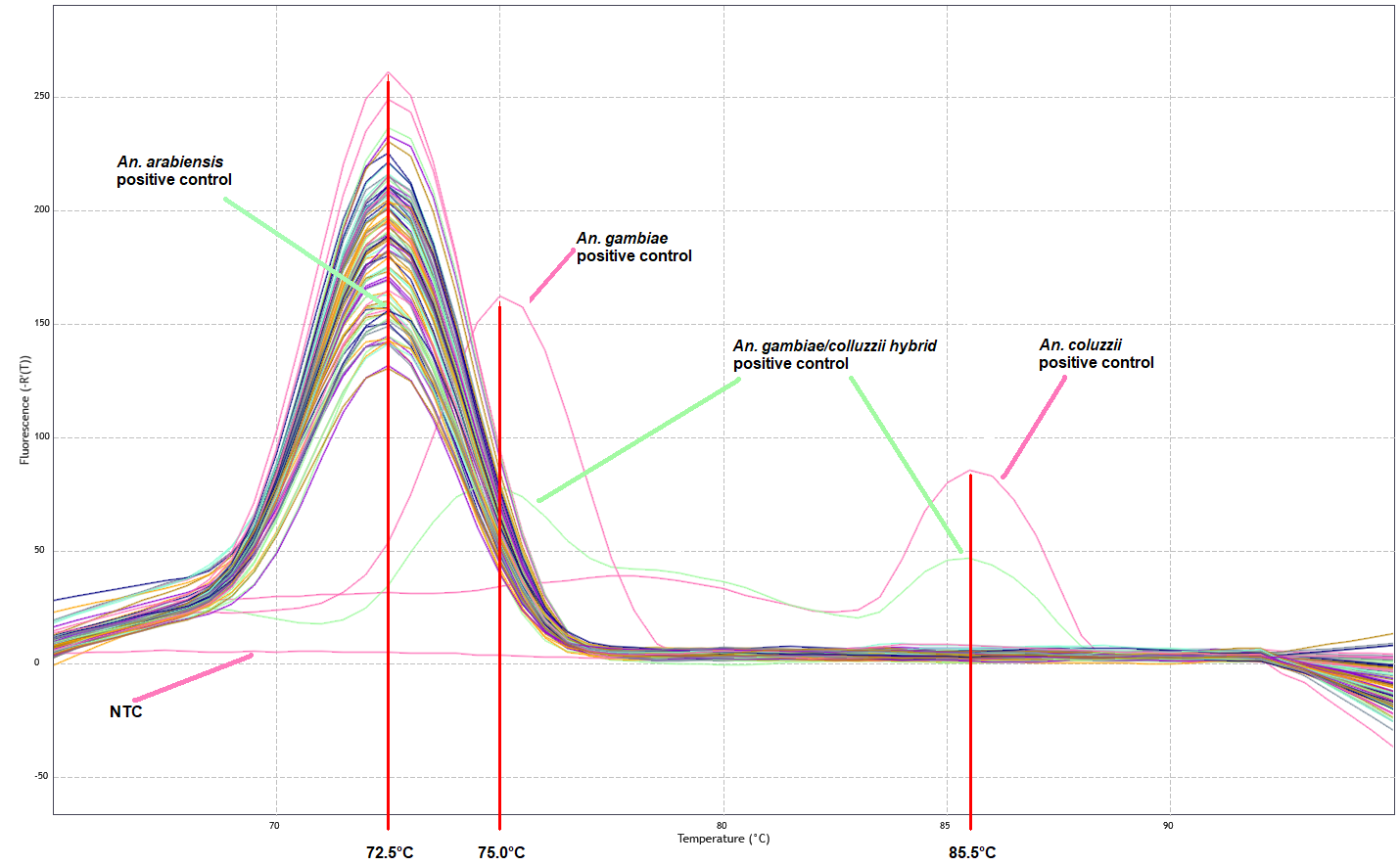

Supplement: S2 Fig — (TIF) [file pone.0215669.s002.TIF]

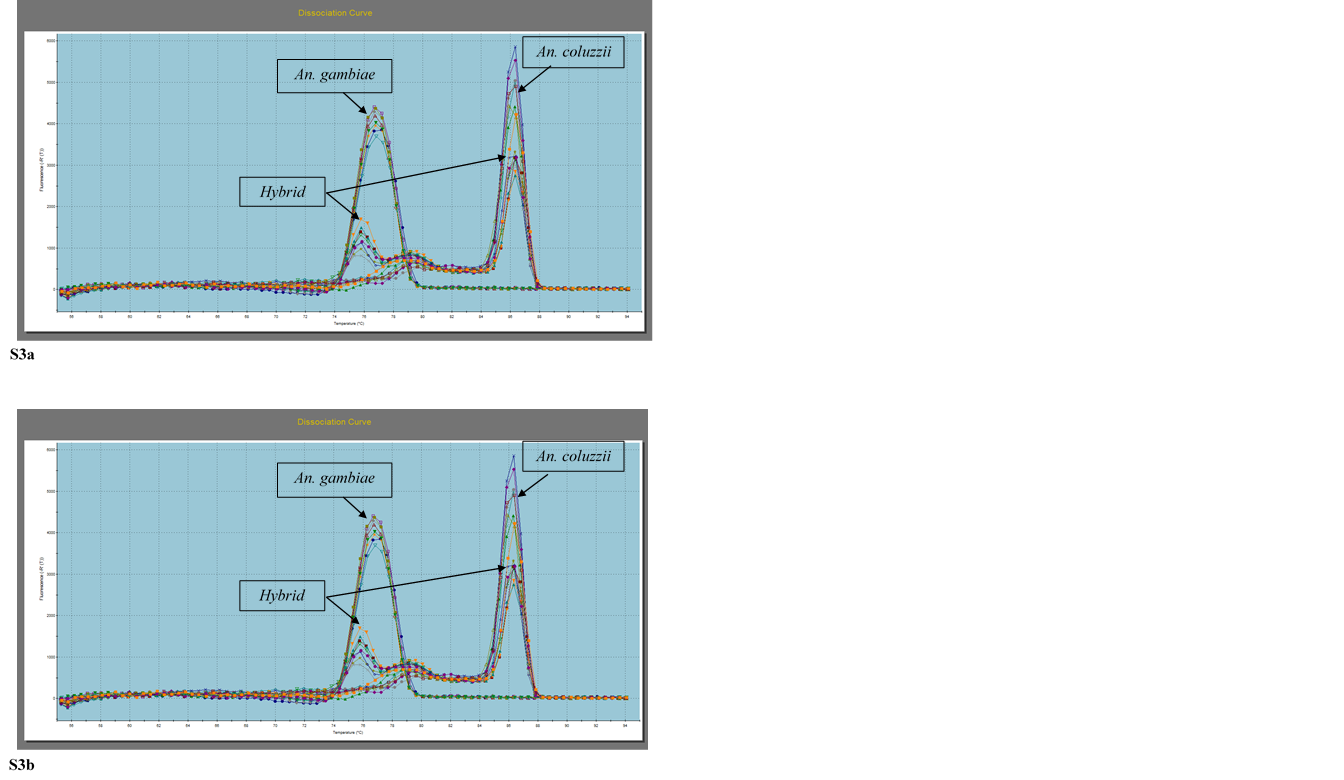

Supplement: S3 Fig — A: Dissociation curves using Brilliant III Ultra-Fast SYBR Green Low ROX QPCR Master Mix. B: Dissociation curves using Luna Universal qPCR Master Mix “Any difference was observed using the same machine and different SYBR green master mix”. (TIF) [file pone.0215669.s003.tif]

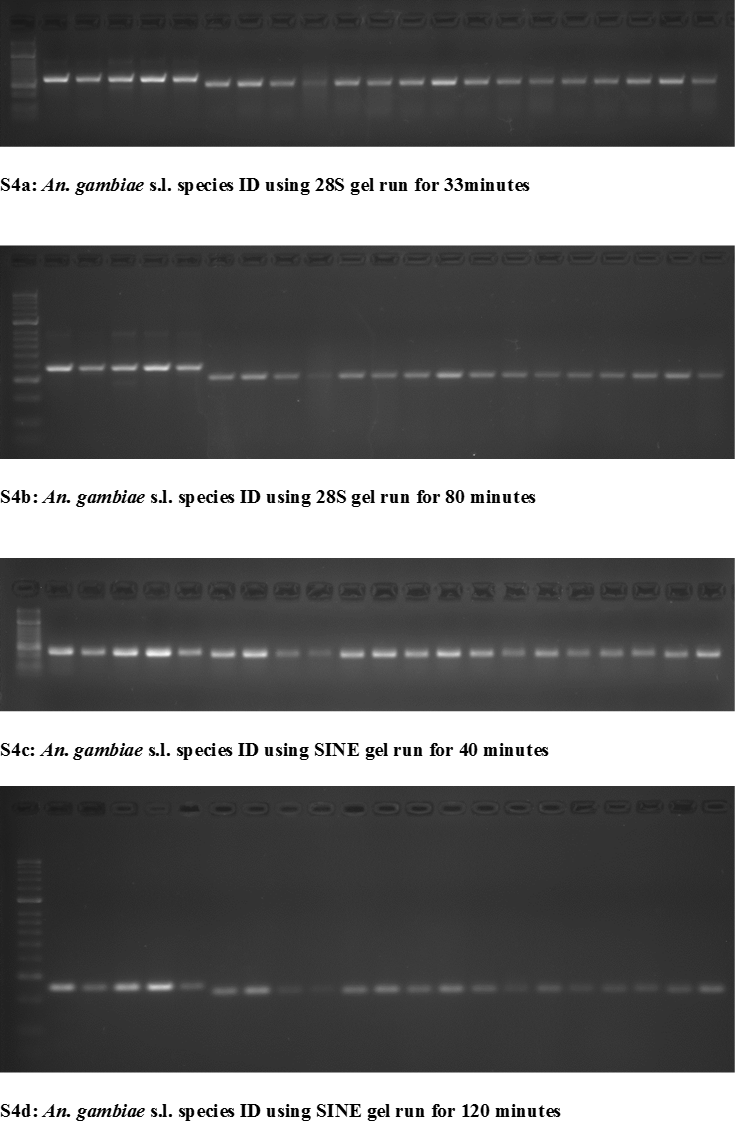

Supplement: S4 Fig — “Samples with inconsistent species ID scores between the 28S gel analysis and the melt-curve technique were re-examined using both the SINE method and the 28S methods. A subset of 24 out of 373 potentially misidentified An. arabiensis, and all 10 potentially misidentified An. gambiae were included. The gel pictures presented here show 5 An. gambiae (Line 1–5) and 16 An. arabiensis (Line 6–21) according to the melt-curves (but scored different previously). Pictures were taken at two intervals; 33 minutes and 80 minutes for the 28S PCRs and 40 and 120 minutes for the SINE200 PCRs (electrophoresis at 120 volts). Size differences are very similar for An. gambiae and An. arabiensis at the shorter run times, making it difficult to reliably assign species ID. The longer gel runs confirm the melt-curve species ID”. (TIF) [file pone.0215669.s004.tif]
