# Supplementary material for: Rapid high throughput SYBR green assay for identifying the malaria vectors Anopheles arabiensis, Anopheles coluzzii and Anopheles gambiae s.s. Giles
Source: PLoS One. 2019 Apr 19;14(4):e0215669. doi: 10.1371/journal.pone.0215669 (PMC6474623; doi:10.1371/journal.pone.0215669)
Supplement: S1 Text — (DOCX) [file pone.0215669.s005.docx]

The following statement is to be used in Microsoft Excel:

**IF(AND(A1="N/A",B1="N/A"),"N/A",IF(AND(A1>85,B1="N/A"),"AC",IF(AND(A1>85,B1>74),"HY",IF(AND(A1>74,B1="N/A"),"AG",IF(AND(A1>71,B1="N/A"),"AA"))))).**

With cell A1 containing the first melt peak and B1 (only present in case of a hybrid) the second melt peak.

The peak temperature criteria are: >85°C = AC = *An. coluzzii*, between 85°C and 74°C = AG = *An. gambiae*, <74°C = AA = *An. arabiensis*, both >85°C and between 85°C and 74°C = HY = *An. gambiae* /*An. coluzzii* hybrid.
